# Liquid biopsy mutation panel for non-small cell lung cancer: analytical validation and clinical concordance

**DOI:** 10.1038/s41698-020-0118-x

**Published:** 2020-06-24

**Authors:** Lee S. Schwartzberg, Hidehito Horinouchi, David Chan, Sara Chernilo, Michaela L. Tsai, Dolores Isla, Carles Escriu, John P. Bennett, Kim Clark-Langone, Christer Svedman, Pascale Tomasini, Oncotype SEQ® Study Investigators and Program Team, Gregory Alexander, Frederick L. Baehner, Thomas Bauer, Anna Bergamaschi, John Crown, Deborah Davison, David A. Eberhard, Nashat Gabrail, James Han, William Irvin, Margarita Lopatin, James Orsini, Bradley T. Sumrall

**Affiliations:** 1 West Cancer Center & Research Institute, 7945 Wolf River Boulevard, Germantown, TN 38138 USA; 2 National Cancer Center Hospital, 5-1-1 Tsukiji, Chuo-ku, Tokyo, 104-0045 Japan; 3 Cancer Care Associates TMPN (now Hunt Cancer Center), 3285 Skypark Dr, Torrance, CA 90505 USA; 4 Instituto Nacional del Tórax, José Manuel Infante 717, segundo piso, Providencia, 7500691 Santiago, Chile; 5 Virginia Piper Cancer Institute, Minnesota Oncology, 800 E 28th St., Suite 602, Minneapolis, MN 55407 USA; 6 University Hospital Lozano Blesa, Avda. S. Juan Bosco no 15, 50009 Zaragoza, Spain; 7 The Clatterbridge Cancer Centre, Clatterbridge Road, Bebington, Wirral, CH63 4JK UK; 8 Genomic Health, Inc. (now Exact Sciences Corp.), 301 Penobscot Dr, Redwood City, CA 94063 USA; 9 Assistance Publique Hôpitaux de Marseille, and Centre de Recherche en Cancérologie de Marseille, Inserm UMR1068, CNRS UMR7258, Aix-Marseille Université, UM105 Marseille, France; 10 Jersey Shore University Hospital, Hackensack Meridian Health, 1945 Route 33, Neptune, NJ 07753 USA; 11 St. Vincent’s University Hospital, Elm Park, Merrion Rd, Dublin 4, Ireland; 12 Gabrail Cancer Center, 4875 Higbee Ave NW., Canton, OH 44718 USA; 13 Bon Secours Cancer Institute at St. Francis, 14051 St. Francis Blvd., Suite 2210, Midlothian, VA 23114 USA; 14 Essex Oncology of North Jersey, 1 Clara Maass Drive Suite 200, Belleville, NJ 07109 USA; 15 Central Georgia Cancer Care, 800 1st St., Suite 410, Macon, GA 31201 USA

**Keywords:** Cancer genomics, Cancer genomics

## Abstract

Molecular testing for genomic variants is recommended in advanced non-small cell lung cancer (NSCLC). Standard tissue biopsy is sometimes infeasible, procedurally risky, or insufficient in tumor tissue quantity. We present the analytical validation and concordance study of *EGFR* variants using a new 17-gene liquid biopsy assay (NCT02762877). Of 144 patients enrolled with newly diagnosed or progressive stage IV nonsquamous NSCLC, 140 (97%) had liquid assay results, and 117 (81%) had both *EGFR* blood and tissue results. Alterations were detected in 58% of liquid samples. Overall tissue-liquid concordance for *EGFR* alterations was 94.0% (95% CI 88.1%, 97.6%) with positive percent agreement of 76.7% (57.7%, 90.1%) and negative percent agreement of 100% (95.8%, 100%). Concordance for *ALK* structural variants was 95.7% (90.1%, 98.6%). This assay detected alterations in other therapeutically relevant genes at a rate similar to tissue analysis. These results demonstrate the analytical and clinical validity of this 17-gene assay.

## Introduction

Improved understanding of the molecular basis of cancer has enabled the personalized treatment of patients with agents targeting cancer-specific gene alterations. For example, in non-small cell lung cancer (NSCLC), targeted therapies are currently approved and preferred over cytotoxic chemotherapy for patients with sensitizing *EGFR* mutations^1–4^, *ALK* and *ROS1* gene rearrangements^5–8^, and *BRAF* variants^9^. There are additional alterations in other genes (*ERBB2*, *RET*, and *MET)* with available targeted therapies and emerging evidence that are included in treatment guidelines^10^, including *NTRK* fusions^11^. The approval of osimertinib for patients with emergence of *EGFR* T790M resistance mutations highlights the growing importance of assessing and targeting emerging mutations associated with sensitivity or resistance^12^. Assessment of patient tumor genomic alteration status, at diagnosis and throughout the course of metastatic disease, is now necessary for optimal therapy.

Even though mutation assessment for *EGFR*, *ALK*, and *ROS1* has been standard of care for several years, testing of patients with advanced disease is suboptimal; reported proportions of patients without *EGFR* testing surpass 25% in some countries^13,14^. In addition, only a low proportion of patients have mutation test results available at their first oncology consultation, resulting in delayed initiation of therapy or initiation of chemotherapy before diagnostic test results are available^13^. The causes of suboptimal mutation assessment are multifactorial, including patient comorbidities, complications of lung biopsy such as bleeding, pneumothorax, and infection, and frequently inadequate sampling leading to costly rebiopsy in about a third of patients^15,16^. Furthermore, increasing understanding of tumor heterogeneity calls into question the representativeness of a biopsy of a single metastatic site^17,18^. Thus, alternative, less invasive methods to assess the genomic alterations of tumors are warranted.

Circulating cell-free DNA (cfDNA) from blood can be used to detect tumor-specific genomic alterations in the metastatic setting in various tumor types, and current sequencing technologies allow for rapid identification of a large number of genomic alterations from a modest volume of blood^19,20^. In addition to reducing discomfort for the patient, assessing genomic alterations in blood versus tumor tissue may more accurately reflect the mutational landscape of metastatic lesions at different sites and the molecular heterogeneity among various tumor sites and regions. Recent data also indicate that liquid biopsy may be useful for monitoring the development of alterations associated with acquired resistance, a potential that carries implications for treatment decisions^21,22^.

The reported concordance of assessing genomic alterations in blood versus in tumor tissue varies widely due to multiple factors, such as the methods used—including analytical platform, varying tumor burden (lower sensitivity in patients with lower tumor burden and earlier stages of disease), and genomic coverage of the regions of interest. It is therefore of clinical importance to characterize the analytical assay performance in the intended use population by assessing the concordance of key actionable genomic alterations detected in plasma with those found in tissue (biopsy/cytology/excision), the current standard of care. The ideal plasma ctDNA mutation panel assay would be prospectively validated in the intended use population and provide clinically actionable results for all genomic mutations that are associated with benefit from targeted therapies (that are FDA-approved, in ASCO, CAP, NCCN guidelines, or in late-stage clinical development with evidence of efficacy and safety).

Here we report the analytical validation of a 17-gene next-generation sequencing (NGS) liquid biopsy panel that assesses genomic alterations in plasma, including validated alterations that, when present in the tumor, may portend benefit from targeted agent treatment approved by regulatory authorities. The panel also assesses select genes that are targets of therapies in late-stage clinical trials and genomic alterations that may be relevant for treatment selection but are supported by preliminary or preclinical evidence only.

We conducted a prospective study assessing EGFR variant concordance between liquid and tissue biopsy in patients with nonsquamous NSCLC for whom the clinical utility of a liquid biopsy-based mutation assessment is high. Many such patients may have tumors that are difficult to biopsy or that yield poor-quality biopsies but for whom knowing the status of genomic alterations is necessary to select optimal therapy. We present here the interim analysis of the concordance study.

## Results

### Analytical validation

Detection thresholds were set to ensure >99% per-sample specificity. The lowest target amount for 95% detection rate (i.e., limit of detection or LOD95) was determined for each variant type and were as follows: insertions/deletions (indels), 0.1% allelic fraction (AF); single-nucleotide variants (SNVs), 0.37% AF; structural variants (SVs), 0.44% AF; and copy number variants (CNVs), ≥3 copies. Using a combination of reference standards and samples shown to harbor a mutation by digital droplet polymerase chain reaction, the positive percent agreement (PPA) and technical positive predictive value were both 98.9%. In the repeatability and reproducibility study, all expected variants were observed, indicative of 100% PPA and reproducibility. The detailed results on specificity, sensitivity, accuracy, repeatability and reproducibility, interfering substances, stability, contamination, and cross talk from the analytical validation are provided in the Supplementary Tables 1–12 and Supplementary Notes.

### Clinical concordance study

The clinical concordance study enrolled 157 patients from 16 sites between April 2016 and October 2017. Thirteen patients were excluded due to failure to satisfy eligibility criteria, including absence of tissue samples, presence of treatment between tissue and liquid biopsies, withdrawal of consent, or death prior to blood draw. The vast majority (140/144) of protocol-eligible patients had evaluable liquid assay results including 121 cohort A patients (those with newly diagnosed metastatic disease or progressive disease on any-line non-*EGFR*-targeted therapy) and 19 cohort B patients (those with progressive disease on *EGFR*-targeted therapy). Four samples were excluded due to blood preprocessing issues; there were no laboratory failures. In cohort A, 117 patients had concurrent *EGFR* tissue results, with a median of 27 days (interquartile range 14–35) between tissue and blood collection.

Patient demographics and clinical characteristics are presented in Table 1. In cohort A, which was used for the primary analysis of tissue/liquid concordance for *EGFR* mutational status, 61% of patients were white and 50% female. The average age was 66 years (range 42–94 years). A large majority of these patients (93%) were newly diagnosed with stage IV NSCLC. Nearly half (48%) of patients had two or more organs with metastases and about three-fourths (74%) had two or more metastatic lesions in all organs.

**Table 1:** Patient demographics and clinical characteristics for the clinical concordance study.

	Cohort A (*n* = 121)	Cohort B (*n* = 19)
Age, years		
Median (range)	66 (42–94)	67 (45–85)
Female	61 (50.4%)	10 (52.6%)
Ethnicity		
Not Hispanic or Latino	91 (88.3%)	12 (92.3%)
Hispanic or Latino	3 (2.9%)	1 (7.7%)
Not reported	9 (8.7%)	0 (0.0%)
Race		
White	74 (61.2%)	9 (47.4%)
Asian	14 (11.6%)	4 (21.1%)
Black or African American	8 (6.6%)	0 (0.0%)
Not reported	25 (20.7%)	6 (31.6%)
Progression status at enrollment		
Newly diagnosed with stage IV disease	113 (93.4%)	0 (0.0%)
Progressing on treatment	8 (6.6%)	19 (100%)
Number of months from diagnosis to progression		
Median (range)	18 (4–53)	15 (2–79)
Nonsquamous NSCLC subtype		
Adenocarcinoma	117 (96.7%)	19 (100%)
Large cell carcinoma	3 (2.5%)	0 (0.0%)
Other	1 (0.8%)	0 (0.0%)
Number of organs with metastases		
1	57 (47.1%)	6 (31.6%)
2 or more	58 (47.9%)	12 (63.2%)
LN metastases only	6 (5.0%)	1 (5.3%)
Number of total metastatic lesions in all organs		
1	32 (26.4%)	4 (21.1%)
2 or more	89 (73.6%)	15 (78.9%)
Country		
France	18 (14.9%)	6 (31.6%)
Ireland	0 (0.0%)	1 (5.3%)
Japan	5 (4.1%)	3 (15.8%)
Spain	27 (22.3%)	7 (36.8%)
United Kingdom	7 (5.8%)	0 (0.0%)
United States of America	64 (52.9%)	2 (10.5%)

*LN* lymph node, *NSCLC* non-small cell lung cancer.

**Fig. 1:**
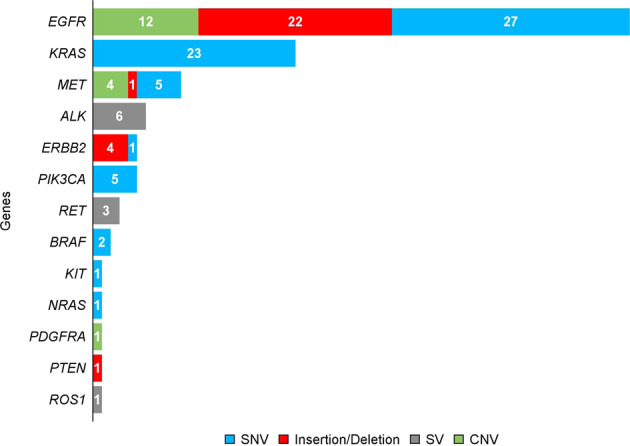
Number of alterations by gene reported in the liquid biopsy assay for 140 patients in cohorts A and B. CNV copy number variant, SNV single-nucleotide variant, SV structural variant.

**Fig. 2:**
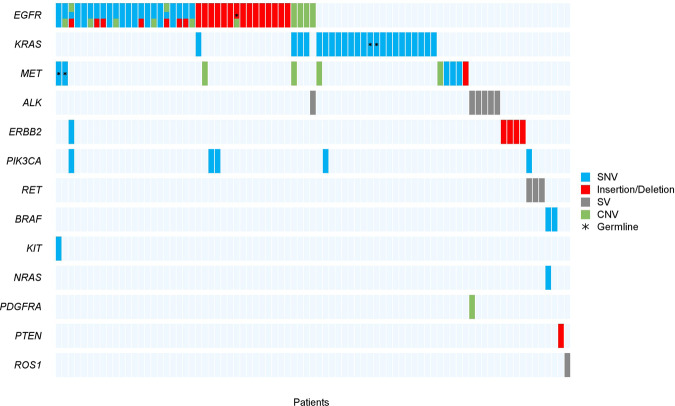
Alterations detected in liquid biopsy in cohorts A and B, by gene and patient (*n* = 81 patients with at least one alteration detected). Each row represents a gene and each column represents an individual patient. CNV copy number variant, SNV single-nucleotide variant, SV structural variant.

Alterations reported by the liquid biopsy assay are presented in Figs. 1 and 2. Overall, the liquid biopsy assay identified 120 alterations across 13 genes in 81/140 (58%) patients, including *EGFR* (29%), *KRAS* (16%), *MET* (7%), *ALK* (4%), *ERBB2* (4%), *RET* (2%), *BRAF* (1%), and *ROS1* (<1%). More than half (54%) of alterations were SNVs, 23% indels, 14% CNVs, and 8% SVs. Most (51/81) patients had only one alteration reported.

In cohort A, the liquid biopsy assay identified 90 genomic alterations in 66 (55%) patients. Among the 90 alterations were 34 *EGFR* variants, which included 13 exon 19 deletions and 14 SNVs. Ten of the *EGFR* SNVs are included in the primary analysis (L858R [*n* = 6], G719A [*n* = 2], L861Q [*n* = 2]). Four other *EGFR* SNVs were found, including one T790M. In 19 cohort B patients (progressing on erlotinib, gefitinib, or afatinib), the assay detected 30 genomic alterations: 13 *EGFR* SNVs (T790M [*n* = 6], L858R [*n* = 3], G719A [*n* = 1], L861Q [*n* = 1], S768I [*n* = 1], V769L [*n* = 1]), five *EGFR* CNVs, eight *EGFR* deletions, one *EGFR* insertion, and one SNV each in *ERBB2*, *KRAS*, and *PIK3CA*. The prevalence of *EGFR* T790M in patients progressing on *EGFR*-targeted therapy was 32% (95% CI: 13–57%).

In the primary concordance analysis of 117 cohort A patients with evaluable tissue and liquid results, there was substantial variation in the tissue tests used (Supplementary Table 13) and the methodology: polymerase chain reaction (52.1%), NGS (32.5%), Sanger (14.5%), and restriction fragment length polymorphism (0.9%). Of 157 enrolled patients, the central Clinical Laboratory Improvement Amendments (CLIA) laboratory test (FoundationOne^®^, Foundation Medicine, Cambridge, MA) was ordered in 33 patients. Eleven (33%) of these tests failed due to insufficient tumor, DNA yield, or tumor purity metrics. In some patients, a small amount of residual material was available after local tissue assessment, increasing the risk of failure due to biopsy volume.

Concordance results for alterations in *EGFR* and *ALK* are shown in Table 2. Thirty patients had the prespecified *EGFR* mutations (exon 19 deletions, L858R, L861Q, G719X, and S768I) in tissue and 23 had these mutations in plasma. All patients with *EGFR* alterations detected in plasma had the same alterations detected in tissue, resulting in a PPA of 76.7% (95% CI: 57.7–90.1%) and a negative percent agreement (NPA) of 100% (95.8–100%). The overall concordance was 94.0% (88.1–97.6%). Patients with *EGFR* alterations reported in tissue but not in plasma tended to have lower tumor burden as indicated by lower DNA yields from blood and a lower proportion of patients with evidence of disease in two or more organs (Supplementary Fig. 1).

**Table 2:** Positive percent agreement (PPA), negative percent agreement (NPA), and overall percent agreement (OPA) with 95% confidence intervals for key *EGFR* and *ALK* alterations in cohort A.

Alteration	*n*	PPA	NPA	OPA
EGFR				
Exon 19 deletions, L858R, L861Q, G719X, or S768I	117	76.7% (57.7%, 90.1%)	100.0% (95.8%, 100%)	94.0% (88.1%, 97.6%)
ALK				
Structural variants	115	50.0% (18.7%, 81.3%)	100.0% (96.5%, 100.0%)	95.7% (90.1%, 98.6%)

Ten patients had ALK translocations detected in tissue, five of whom had ALK alterations detected in liquid, with a resulting PPA of 50.0% (18.7–81.3%), NPA of 100.0% (96.5–100.0%), and overall percent agreement (OPA) of 95.7% (90.1–98.6%). In one patient with an alteration detected in tissue only, the liquid biopsy signal for ALK translocation was just below the threshold for calling the variant. The details of concordance for each gene in the panel are presented in Supplementary Table 14. The number of assessments in tumor tissue for some of the genes was limited.

Among patients with key *EGFR* alterations (listed in Table 2) in both tissue and liquid (*n* = 23) the best response (by imaging and/or clinical assessment) in patients treated with targeted therapy was available in 21 patients. According to both imaging and clinical assessment, there was partial response in 11 patients (52%) and stable disease in 10 (48%). In patients with *EGFR* alterations detected in tissue only, the response rate was captured in six of seven patients. Best response (by imaging and/or clinical assessment) was partial response in three patients (50%) and stable disease in three patients (50%).

An ad hoc analysis was performed to examine the association of response rate with AF. In patients with AF below 1%, 2/8 (25%) had partial responses and 6/8 (75%) had stable disease. In patients with an AF of 1% or above, 9/13 (69%) had partial responses and 4/13 (31%) had stable disease.

Best response to first therapy after liquid biopsy was available in five of five patients with *ALK* alterations detected in both tissue and liquid. All patients received *ALK*-targeted therapy. Two patients (40%) had complete responses and two patients had partial responses (40%) by imaging. One patient (20%) had progressive disease by clinical assessment. Among the five patients with *ALK* alterations detected in tissue only, four had best response data available: one had a complete response, one had stable disease, and two had progressive disease.

## Discussion

There are many potential uses of liquid biopsy testing in oncology, from screening of early-stage disease to monitoring of treatment effect. Presently, there is relatively little evidence supporting the utility of such testing for most of these purposes, except for patients with advanced or metastatic disease who are candidates for systemic targeted therapy but in whom tissue testing cannot be performed successfully^23^. Such patients may have insufficient diagnostic tissue for testing or may be difficult to biopsy because of tumor location, comorbidities, bleeding disorders, or a strong personal preference. Even in these patients, only limited data from prospective studies exist to support use of tests assessing actionable genomic alterations across multiple genes, despite a high unmet need. Data also support liquid biopsy assessment in patients progressing on targeted therapy where there are identified acquired mutations associated with resistance and sensitivity to other drugs and where the patient is challenging to biopsy^21,22^.

In previously reported prospective clinical concordance studies, the sensitivity and specificity for detecting *EGFR* mutations in liquid versus tissue was similar to that reported for the approved Cobas *EGFR* test^24–26^. The analytical validation of the liquid biopsy assay described herein demonstrated high sensitivity, specificity, and reproducibility for detecting SNVs, indels, SVs, and CNVs. Importantly, there were no *EGFR* alterations found in liquid that were not present in tumor tissue in newly diagnosed patients, leading to an NPA of 100%. The PPA below 80% supports reflex tumor tissue testing when a liquid test is negative, similar to the Cobas *EGFR* test. The OPA for all SNVs, indels, and CNVs in the panel was comparable with that for *EGFR* (SNV 89.7% [82.8–94.6%], indels 95.7% [90.3–98.6%], CNVs 90.3% [74.2–98.0%]). The OPA for translocations was also similar to *EGFR*, while the PPA based on 14 positive patients was somewhat lower (50% [23.0–77.0%]). Gene rearrangements can be more challenging to identify than SNVs or small indels when using hybrid capture versus amplicon-based enrichment methodologies. It is thus possible that the true concordance between cfDNA and tissue indeed may be lower for gene rearrangements than for SNVs when using hybrid capture technologies.

Overall, the data across many studies using a variety of modern methods demonstrate sensitivities for *EGFR* alterations assessed in liquid biopsy versus tumor tissue to be in the 70–85% range in patients with metastatic disease^24–36^. The consistency of our results with published data suggests that this sensitivity limit is driven in part by biological constraints, perhaps disparity in overall tumor burden or differences in DNA shedding from tumor cells in various tissue locations. Our planned analysis by tumor burden revealed that failure to detect alterations in liquid was associated with lower tumor burden, as indicated by lower DNA yields from blood and evidence of disease in fewer than two organs. These findings are consistent with the literature^33^. It is important to recognize that the theoretical biological limit of sensitivity to detect tumor mutations is a function of both tumor mutant AF and the total amount of DNA. While some assays, in principle, may have an LOD95 of 0.001% using contrived samples containing more genome equivalents than present in a typical patient sample, it is unlikely that this can be consistently achieved in clinical samples. Thus, samples containing a representative number of genome equivalents are recommended when establishing LOD95. The ability to make correct variant calls at very low AF is important, as recent evidence shows that patients with very low variant AFs may have excellent responses to targeted therapy^37^. In our study, although the number of patients with an AF below 1% was small, a high proportion of these had partial response or stable disease consistent with benefit from *EGFR*-directed therapy.

The results from the cohort of patients progressing on erlotinib, gefitinib, or afatinib demonstrate that the assay can be used to detect alterations associated with resistance/sensitivity in patients progressing on therapy. The reported rate of *EGFR* T790M of 32% is on the lower end of that reported in the literature based on tumor tissue testing^38–40^, possibly reflecting the small sample size or the high proportion of Japanese patients in this cohort (the T790M mutation rate in Japanese patients may be lower than in Caucasians)^41^. In addition, the cohort is somewhat enriched in *EGFR*-mutant patients due to the participation of a Japanese site, as well as sites that are referral centers for *EGFR-*mutant patients. The patient population is thus not completely representative of an all-comer patient population in either Caucasians or East Asians, but the enrichment of *EGFR*-mutant patients was important in order to make study accrual feasible.

The FDA recently approved a broad tumor tissue mutation panel^42^, which will likely increase the use of broad mutational profiling. However, the sample requirements for broad panel testing can lead to high test failure rates in small samples: over 28% for endoscopic biopsies and 51% for fine-needle aspirates^43^. This is an especially important issue in lung cancer, where there are many actionable alterations yet the NGS test failure rate is over 26%^43^. This could create an increased need for rebiopsy, a possible shift toward larger biopsies with higher risks of adverse events, or an alternative assessment by liquid biopsy. In our study, the clinical outcome captured in patients mutation-positive by liquid biopsy is consistent with what has been reported for patients mutation-positive by tumor tissue biopsy. Given 100% NPA and a PPA of less than 80% for the liquid biopsy assay, a negative liquid biopsy should trigger a reflex tumor tissue biopsy if feasible. It should be emphasized that tissue biopsy still has the advantage of providing diagnostic/histological information and suitable material for the assessment of *PD1*/*PDL1* status and other histologic assessments, such as tumor-infiltrating lymphocytes. Given current guidelines that strongly recommend first-line treatment with targeted agents rather than immunochemotherapy for patients with sensitizing alterations in *EGFR*, *ALK*, *ROS1*, and *BRAF*^10^, an initial liquid biopsy assessment may be medically appropriate in many patients and could potentially reduce delays in initiation of treatment that cause anxiety in patients^44^. Patient preference regarding liquid biopsy versus tissue biopsy is not well documented but is a field where research is warranted, as it seems likely that many patients would strongly prefer a less invasive and risky procedure.

There are limitations to the conclusions that can be drawn from this concordance study. While a comparison of liquid biopsy to tumor tissue biopsy results is needed for assay validation, the clinical advantages of liquid biopsy may be most obvious for patients who do not have tissue results, either because of tissue assay failure or because the tumors are challenging to biopsy. Thus, the study population may not be fully representative of a primary target population for clinical use. The large majority of patients in this study had de novo metastatic disease, and it is possible that relapsing patients have a differential expression of liquid versus tissue genomic alterations. Location of the primary tumor and metastatic lesions is likely the most common reason making biopsy difficult and risky. As data in the literature and our results indicate, a lower tumor burden may be associated with a lower sensitivity. In addition, the study permitted local assessment of mutation status in tumor tissue and many different methods were used. Central assessment using an NGS panel was offered for all patients, but only ~30% of patients underwent testing with the central NGS panel. The reasons for this fairly low rate of central testing may include a preference for faster local results or the lack of sufficient residual material after local testing. The heterogeneity of tests and methods used for tumor tissue assessment can be seen as a strength of this study, however, as it reflects current clinical practice. Importantly, that the NPA was 100% and that the concordance was similar when compared with either central NGS tests or local tests indicate that the results were not negatively impacted.

In conclusion, the 17-gene liquid biopsy panel is analytically validated with consistent performance across SNVs, indels, translocations, and CNVs. The concordance with tumor tissue biopsy for clinically relevant alterations in *EGFR* is comparable with that of the approved liquid Cobas *EGFR* test. In addition, the 17-gene panel can detect alterations in other genes relevant for treatment decisions in NSCLC. Liquid biopsy assessment may be clinically helpful in the substantial proportion of patients for whom obtaining a tissue biopsy is challenging, there is a strong patient preference, or tissue biopsy or analysis has failed.

## Methods

The 17-gene liquid biopsy assay was performed in a single CLIA-certified laboratory at Genomic Health, Inc. (Redwood City, CA). Plasma was obtained from whole blood (2 × 10 mL tubes, Cell-Free DNA BCT, Streck) using the standard double spin method; blood was spun for 10 min at 1500 *g* ± 150 *g*, plasma isolated and re-spun at 3000 *g* ± 150 *g* for 10 min, and transferred to a clean tube. Extraction was performed using a proprietary methodology based on the MagMAX Cell-Free DNA Isolation Kit (Thermo Fisher Scientific). DNA quantitation was performed using the Quant-iT™ PicoGreen^®^ dsDNA kit (Thermo Fisher Scientific). To mimic cfDNA, reference control genomic DNA was sheared using the E220 Focused-Ultrasonicator (Covaris) followed by size selection using AMPure XP Beads (Agencourt/Beckman Coulter). The resulting DNA was assessed using the 2100 Bioanalyzer (Agilent) and samples required to be >100 and <200 bp. Whole genome libraries were prepared by a proprietary methodology using the KAPA Hyper Prep Kit (Kapa Biosystems/Roche) and dual-indexed adapters (Integrated DNA Technologies). Resulting pond libraries were quantified and quality controlled using the 4200 TapeStation Instrument (Agilent Genomics). Hybrid capture was performed using a modified SeqCap EZ HyperCap (NimbleGen/Roche) workflow and baits (Integrated DNA Technologies) designed to cover the 17-gene target regions. The genes and alterations covered in the panel are detailed in Table 3. Enriched libraries were quantified using the 4200 TapeStation Instrument (Agilent Genomics).

**Table 3:** Genes/alterations included in the 17-gene liquid biopsy panel.

Gene	Variant type(s) reported
ALK	Targeted and de novo SNVs, targeted and de novo SVs
AR	Targeted and de novo SNVs
BRAF	Targeted and de novo SNVs, targeted and de novo indels
BRCA1	Targeted and de novo SNVs, targeted and de novo indels
BRCA2	Targeted and de novo SNVs, targeted and de novo indels
EGFR	Targeted and de novo SNVs, CNV gains, targeted and de novo indels
ERBB2	Targeted and de novo SNVs, CNV gains, targeted and de novo indels
ESR1	Targeted and de novo SNVs, CNV gains
KIT	Targeted and de novo SNVs, CNV gains, targeted and de novo indels
KRAS	Targeted and de novo SNVs, targeted and de novo indels
MET	Targeted and de novo SNVs, CNV gains, targeted and de novo indels
NRAS	Targeted and de novo SNVs
PDGFRA	Targeted and de novo SNVs, CNV gains, targeted and de novo indels
PIK3CA	Targeted and de novo SNVs, CNV gains, targeted and de novo indels
PTEN	Targeted and de novo indels
RET	Targeted and de novo SNVs, targeted and de novo indels, targeted and de novo SVs
ROS1	Targeted and de novo SNVs, targeted and de novo SVs

The panel includes detection of single-nucleotide variants (SNVs), copy number variant (CNV) gain or loss, targeted insertions/deletions (indels), and targeted structural variants (SVs).

Paired-end sequencing was performed on the HiSeq^®^ 2500 (Illumina). Samples to be sequenced on the same flowcell were pooled together at 7 pM. Clustering was performed using the HiSeq Rapid PE Cluster Kit v2 (Illumina) and performed on the HiSeq^®^ 2500. Samples were sequenced for 101 cycles for both Read 1 and Read 2. PhiX Control (Illumina) was included on each flowcell and used as a sequencing control. For variant calling of SNV and copy number variant (CNV), proprietary continuous metrics based on probabilistic models were employed. Final baseline parameter values for models were derived using data from an independent set of 103 cfDNA samples from healthy volunteers. Indels and SV detection were achieved using proprietary bioinformatic algorithms. In all cases, selection of detection rules and cutoffs was informed by limit of blank (LOB) for each of the various detection metrics, in order to control for specificity. A propriety SNP-signature comparison module was developed to identify potential pre-index cross-contamination or carryover between samples, validated in the Interfering Substances Study and applied to all relevant Analytical Validation studies and Clinical Validation.

A detailed technical description of the analytical validation, including design and statistical analysis, methods for LOB, limit of detection, interfering substances, and accuracy studies is included in the Supplementary File.

### Clinical concordance study

We conducted a global multicenter prospective clinical study (NCT02762877) to characterize the concordance of key clinically relevant genomic alterations in DNA extracted from formalin-fixed, paraffin-embedded tumor tissue (biopsy/excision/cytology) and in cfDNA from liquid biopsy (blood), and the frequencies of genomic alterations identified in liquid biopsy (listed in Table 3) in patients with stage IV nonsquamous NSCLC. Patients seeking treatment at 16 oncology centers in the United States of America, Europe, and Japan who were identified to meet eligibility criteria were enrolled. The study enrolled two cohorts: cohort A, patients who were either newly diagnosed with metastatic disease or progressive disease on non-*EGFR*-targeted therapy (any line); and cohort B, patients with progressive disease on *EGFR*-targeted therapy (erlotinib, gefitinib, afatinib). Full inclusion and exclusion criteria are in Supplementary Table 15. All samples were collected with institutional review board approval (Asentral IRB) and written informed patient consent. Concordance analysis focused on cohort A. Tissue biopsy and blood collection were less than 8 weeks apart with no new systemic antitumoral treatment given in the interval between the tissue biopsy and blood collection (local therapy, such as radiation, was permitted). Tissue analysis by a central CLIA laboratory (Foundation Medicine, Inc.) was offered but not required. The results of the central laboratory assessment were used in the concordance analysis. If no central laboratory result was available, the results from the local assessment of genomic alteration status in tissue were used. In addition, detection of *EGFR* T790M alterations in plasma was characterized in cohort B patients where tissue sample collection was not required. Patients were followed to collect treatment given after liquid biopsy, best response to this treatment (complete response, partial response, stable disease, or progressive disease), and date of clinical or radiological progression for up to 12 months after liquid biopsy. Presence of any of the prespecified clinically actionable *EGFR* alterations (exon 19 deletions, L858R, L861Q, G719X, and S768I) was considered as *EGFR*-positive for the primary analysis. PPA, NPA, and OPA were calculated^45^. Two-sided 95% Clopper–Pearson confidence intervals were reported. The interim analysis was prespecified to occur after enrollment of at least 30 patients with *EGFR* alterations. Individuals involved in laboratory analysis of liquid samples were blinded to clinical data and tissue biopsy results. Data were analyzed using SAS software, version 9.4, of the SAS System for Windows (Copyright 2018 SAS Institute Inc. SAS and all other SAS Institute Inc. product or service names are registered trademarks or trademarks of SAS Institute Inc., Cary, NC, USA).

### Reporting summary

Further information on research design is available in the Nature Research Reporting Summary linked to this article.

## Supplementary information

Supplement

Reporting Summary

## Data Availability

The data that support the findings of the clinical concordance study are available from the corresponding author upon reasonable request. Technical details of the analytical validation study are available in the Supplementary File. Proprietary bioinformatics methods will remain confidential and will not be shared.

## Code availability

The analyses presented in this paper used standard SAS procedures and data steps. No custom code or algorithms were created.
