# Supplementary material for: Liquid biopsy mutation panel for non-small cell lung cancer: analytical validation and clinical concordance
Source: NPJ Precis Oncol. 2020 Jun 24;4:15. doi: 10.1038/s41698-020-0118-x (PMC7314769; doi:10.1038/s41698-020-0118-x)
Supplement: Supplementary file 1 — Supplement [file 41698_2020_118_MOESM1_ESM.pdf]

Supplementary Figures

Supplementary Figure 1. DNA yield in patients with *EGFR* mutations identified in tissue and liquid biopsy and in patients where *EGFR* mutations were identified in tissue only. It should be noted that although yields tended to be lower in patients with alterations identified only in tissue, many patients with alterations in liquid had yields at the same level.

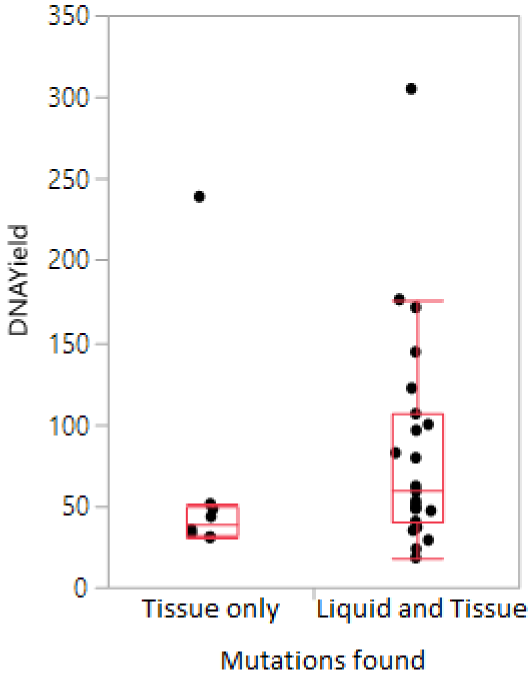

Supplementary Tables

Supplementary Table 1. Study design for establishing SNV, Indel, and SV LOD.

Batch	AF	Input (ng)	# Replicates
1	1	30	5
1	0.7	30	5
1	0.4	30	20
1	0.2	30	20
1	0.05	30	3
2	1.2	30	3
2	0.7	30	5
2	0.5	30	20
2	0.25	30	20
2	0.125	30	3
2	0.07	30	3
3	1.5	30	3
3	0.8	30	5
3	0.6	30	20
3	0.35	30	20
3	0.1	30	4
4	1.3 (SNV) 0.5 (Indel)	10	10
4	1.3 (SNV) 0.5 (Indel)	15	5
4	0.7 (SNV) 0.38 (Indel)	50	5

Supplementary Table 2. Study design for establishing CNV LOD.

Batch	Copies	Input (ng)	# Replicates	CNV
1	3.2	50	5	*MET & EGFR*
1	3	10	10	*ERBB2*
1	3	50	5	*ERBB2*
2	2.6	30	20	*MET & EGFR*
2	3	30	20	*MET & EGFR*
2	3.2	30	5	*MET & EGFR*
2	3	30	20	*MET*
2	2.8	30	18	*MET*

Supplementary Table 3. Run matrix for repeatability and reproducibility study. Pool 1: HD730 and HD-C1109; Pool 2: HD-C1109 and HD782; Pool 3: HD753.

Run	Week	DNA Input (ng)	Adapters Lot #	Bait Reagent Lot #	Pond Reagent Lot #	Pool #	# of Reps	Sequencer Instrument #	Catch Instrument #
1	1	30	1	2	1	1	3	1	1
2	1	30	1	2	1	2	3	1	1
3	1	30	1	2	1	3	3	1	1
4	2	30	2	2	2	1	3	2	2
5	2	30	2	2	2	2	3	2	2
6	2	30	2	2	2	3	3	2	2
7	3	30	1	1	3	1	3	2	1
8	3	30	1	1	3	2	3	2	1
9	3	30	1	1	3	3	3	2	1

Supplementary Table 4. SNV LOD95 point estimates**.**

Gene ID	SNV	Batch 1	Batch 2	Batch 3	Average
KRAS	A146T	0.39	0.27	0.39	0.35
KRAS	G13D	0.16	0.41	0.32	0.3
KRAS	G12S	0.35	0.27	0.45	0.36
NRAS	Q61H	0.23	0.27	0.39	0.3
PIK3CA	E542K	0.47	0.28	0.4	0.38
PIK3CA	H1047R	0.2	0.28	0.39	0.29
MET	Y1253D	0.34	0.31	0.3	0.32
BRAF	V600E	0.23	0.14	0.18	0.18
BRAF	V600M	0.18	0.22	0.28	0.23
EGFR	G719S	0.19	0.29	0.38	0.29
Pooled		0.38 (0.33, 0.40)	0.32 (0.29, 0.35)	0.43 (0.38, 0.45)	0.37

Supplementary Table 5. Indel LOD95 point estimates.

Gene/Indel type	LOD95 Batch 1 (AF)	LOD95 Batch 2 (AF)	LOD95 Batch 3 (AF)	LOD95 Batch Ave (AF)
EGFR 9bp/Ins	0.20%	0.13%	0.07%	0.13%
EGFR 15bp/Del	0.05%	0.07%	0.10%	0.07%
Overall Average				0.10%

Supplementary Table 6. SV LOD95 point estimates.

Structural Variant	LOD95 Batch 1 (AF)	LOD95 Batch 2 (AF)	LOD95 Batch 3 (AF)	LOD95 Batch Ave (AF)
SLC34A2->ROS1	0.59	0.44	0.60	0.54
CCDC6->RET	0.36	0.28	0.48	0.37
ROS1->SLC34A2	0.36	0.36	0.50	0.41
Overall Average				0.44

Supplementary Table 7. CNV LOD95 point estimates.

**Gene**	**LOD95**
EGFR and *MET*	≤3 Copies
ERBB2	≤3 Copies
MET	≤3 Copies

Supplementary Table 8. Detailed information for the 81 samples used in accuracy assessment that passed the standard quality metrics.

Sample Type	Sample Source	N	Notes
Patient blood	Conversant Bio	35	Purchased patient specimens with known mutation in FFPE sample. Accuracy established by ddPCR using cfDNA and/or pond library
	LSv1.0 Physician Experience Program	1	*EML4-ALK* fusion (detected by LSv1.0)
Ref standard/ cell line	HorizonDX Reference Standards	25	Used Vendor provided AF to dilute
	Cancer cell line	5	CNV only
	Variant Negative Control	15	A total of 5 HapMap samples, each sheared and size-selected in 3 independent batches

Supplementary Table 9. Accuracy results.

# Unique variant/sample combinations		Comparator	
		Variant Detected (+)	Variant Not Detected (-)
17-gene liquid biopsy assay	Variant Detected (+)	174	2
	Variant Not Detected (-)	2	NA*

*Given that the 17-gene liquid biopsy assay can detect de novo SNVs and Indels, the potential number of variants not detected (or comparator) is almost impossible to compute, and therefore the number of "true negatives" is not applicable.

Supplementary Table 10. PPA and TPPV.

	Estimates	Jeffrey 95% lower bound	Clopper-Pearson 95% lower bound
PPA	98.9%	96.4%	96.0%
TPPV	98.9%	96.4%	96.0%

Supplementary Table 11. Summary of additional unexpected variants in HapMap DNA (Variant Negative Control DNA) and confirmed by ddPCR.

Sample	Variant ID	Variant type	Observed AF	Detected by 17-gene liquid biopsy assay	Detected by ddPCR assay	Notes
NA24361	7:55220276:C:T	de novo SNV	0.64%	YES	YES	*Real mutation as confirmed by ddPCR*
			0.67%	YES	YES	
			1.20%	YES	YES	
NA24361	17:37883175:C:A	de novo SNV	0.78%	YES	YES	*Real mutation as confirmed by ddPCR*
			1.00%	YES	YES	
			0.94%	YES	YES	

Supplementary Table 12. Frequency of observations as number of actual observations over number of expected observations.

	Week 1			Week 2			Week 3		
**Variant**	**Pool 1**	**Pool 2**	**Pool 3**	**Pool 1**	**Pool 2**	**Pool 3**	**Pool 1**	**Pool 2**	**Pool 3**
SNV (CHR/POS)									
BRAF (7/140453137)	3/3			3/3			3/3		
BRAF (7/140453136)	3/3		3/3	3/3		3/3	3/3		3/3
NRAS (1/115256528)	3/3			3/3			3/3		
PIK3CA (3/178936082)	3/3			3/3			3/3		
PIK3CA (3/178952085)	3/3		3/3	3/3		3/3	3/3		3/3
PIK3CA (3/178936091)			3/3			3/3			3/3
KRAS (12/25378562)	3/3			3/3			3/3		
KRAS (12/25398281)	3/3			3/3			3/3		
KRAS (12/25398285)	3/3			3/3			3/3		
EGFR (7/55241707)	3/3			3/3			3/3		
MET (7/116423428)	3/3			3/3			3/3		
Indel									
EGFR deletion	3/3		3/3	3/3		3/3	3/3		3/3
EGFR insertion		3/3	3/3		3/3	3/3		3/3	3/3
SV									
CCDC6/*RET*	3/3	3/3	3/3	3/3	3/3	3/3	3/3	3/3	3/3
SLC34A2/*ROS1*		3/3	3/3		3/3	3/3		3/3	3/3
ROS1/*SLC34A2*		3/3	3/3		3/3	3/3		3/3	3/3
CNV									
MYC			3/3			3/3			3/3

Supplementary Table 13. Local tests used in tissue analysis for the clinical concordance study.

Test Name	No. (%)
Qiagen Therascreen EGFR	23 (19.7%)
FoundationOne	22 (18.8%)
Sanger sequencing	17 (14.5%)
Roche Cobas EGFR Mutation Test v2	13 (11.1%)
Taqman PCR	9 (7.7%)
Caris Life Sciences 592 Gene Panel	7 (6.0%)
Hopital Nord Custom PCR	7 (6.0%)
SNaPShot multiplex PCR	5 (4.3%)
OnkoSight NGS EGFR sequencing	2 (1.7%)
PathGroup NGS	2 (1.7%)
Torrent Ion S5 High Flow Sequencing	2 (1.7%)
Ascend Genomics PCR	1 (0.9%)
Biocartis Idylla EGFR mutation cartridge	1 (0.9%)
Caris Life Sciences RFLP	1 (0.9%)
Mayo Clinic Lung Panel	1 (0.9%)
Oncomine Focus Assay	1 (0.9%)
PCR-Invader Assay	1 (0.9%)
Paradigm Cancer Diagnostic	1 (0.9%)
Scorpions ARMS	1 (0.9%)

Supplementary Table 14. Concordance by gene and variant for the clinical concordance study. Percentages for percent positive agreement (PPA), negative percent agreement (NPV), and overall percent agreement (OPA) are given with two-sided 95% Clopper-Pearson confidence intervals in parentheses. TP = true positive (variant found in both liquid and tissue), FP = false positive (variant found in liquid but not tissue), FN = false negative (variant found in tissue but not liquid), TN = true negative (variant not found in liquid or tissue).

Gene	Variant	TP	FP	FN	TN	Total	% in tissue	PPA	NPA	OPA
ALK	Rearrangement	5	0	5	105	115	9%	50.0% (18.7%,81.3%)	100.0% (96.5%,100.0%)	95.7% (90.1%,98.6%)
EGFR	Exon 19 deletions, L858R, L861Q, G719X or S768I	23	0	7	87	117	26%	76.7% (57.7%,90.1%)	100.0% (95.8%,100.0%)	94.0% (88.1%,97.6%)
EGFR	Exon 19 deletions	13	0	2	102	117	13%	86.7% (59.5%,98.3%)	100.0% (96.4%,100.0%)	98.3% (94.0%,99.8%)
EGFR	Exon 20 insertions	0	0	3	94	97	3%	0.0% (0.0%,70.8%)	100.0% (96.2%,100.0%)	96.9% (91.2%,99.4%)
EGFR	Any exon 19 del or p.L858R	19	0	5	93	117	21%	79.2% (57.8%,92.9%)	100.0% (96.1%,100.0%)	95.7% (90.3%,98.6%)
EGFR	Any SNV	10	0	5	102	117	13%	66.7% (38.4%,88.2%)	100.0% (96.4%,100.0%)	95.7% (90.3%,98.6%)
EGFR	p.G719A	2	0	2	112	116	3%	50.0% (6.8%,93.2%)	100.0% (96.8%,100.0%)	98.3% (93.9%,99.8%)
EGFR	p.S768I	0	0	1	98	99	1%	0.0% (0.0%,97.5%)	100.0% (96.3%,100.0%)	99.0% (94.5%,100.0%)

Gene	Variant	TP	FP	FN	TN	Total	% in tissue	PPA	NPA	OPA
EGFR	p.L858R	6	0	3	108	117	8%	66.7% (29.9%,92.5%)	100.0% (96.6%,100.0%)	97.4% (92.7%,99.5%)
EGFR	p.L861Q	2	0	0	114	116	2%	100.0% (15.8%,100.0%)	100.0% (96.8%,100.0%)	100.0% (96.9%,100.0%)
KRAS	SNVs	15	0	3	36	54	33%	83.3% (58.6%,96.4%)	100.0% (90.3%,100.0%)	94.4% (84.6%,98.8%)
BRAF	SNV	1	0	1	30	32	6%	50.0% (1.3%,98.7%)	100.0% (88.4%,100.0%)	96.9% (83.8%,99.9%)
KIT	SNV	0	0	2	20	22	9%	0.0% (0.0%,84.2%)	100.0% (83.2%,100.0%)	90.9% (70.8%,98.9%)
MET	SNV	1	0	2	19	22	14%	33.3% (0.8%,90.6%)	100.0% (82.4%,100.0%)	90.9% (70.8%,98.9%)
PIK3CA	SNV	1	0	0	21	22	5%	100.0% (2.5%,100.0%)	100.0% (83.9%,100.0%)	100.0% (84.6%,100.0%)
NRAS	SNV	1	0	0	21	22	5%	100.0% (2.5%,100.0%)	100.0% (83.9%,100.0%)	100.0% (84.6%,100.0%)
ROS1	Rearrangement	1	0	2	69	72	4%	33.3% (0.8%,90.6%)	100.0% (94.8%,100.0%)	97.2% (90.3%,99.7%)
KIF5B-RET	Rearrangement	1	0	0	22	23	4%	100.0% (2.5%,100.0%)	100.0% (84.6%,100.0%)	100.0% (85.2%,100.0%)
ERBB2	Insertion/ deletion	4	0	0	34	38	11%	100.0% (39.8%,100.0%)	100.0% (89.7%,100.0%)	100.0% (90.7%,100.0%)

Gene	Variant	TP	FP	FN	TN	Total	% in tissue	PPA	NPA	OPA
MET	Insertion/ deletion	0	0	0	22	22	0%		100.0% (84.6%,100.0 %)	100.0% (84.6%,100.0 %)
PTEN	Insertion/ deletion	0	0	0	22	22	0%		100.0% (84.6%,100.0 %)	100.0% (84.6%,100.0 %)
MET	Amplification	1	1	0	29	31	3%	100.0% (2.5%,100.0 %)	96.7% (82.8%,99.9 %)	96.8% (83.3%,99.9 %)
EGFR	CNV	2	2	0	25	29	7%	100.0% (15.8%,100.0 %)	92.6% (75.7%,99.1 %)	93.1% (77.2%,99.2 %)
PDGFRA	CNV	0	0	0	22	22	0%		100.0% (84.6%,100.0 %)	100.0% (84.6%,100.0 %)
EGFR, KRAS, BRAF, KIT, MET, NRAS, PIK3CA	Any SNV	28	0	12	77	117	34%	70.0% (53.5%,83.4 %)	100.0% (95.3%,100.0 %)	89.7% (82.8%,94.6 %)
EGFR, ERBB2, MET, PTEN	Any insertion/ deletion	17	0	5	95	117	19%	77.3% (54.6%,92.2 %)	100.0% (96.2%,100.0 %)	95.7% (90.3%,98.6 %)
ALK, ROS1, RET	Any rearrangement s	7	0	7	10 1	115	12%	50.0% (23.0%,77.0 %)	100.0% (96.4%,100.0 %)	93.9% (87.9%,97.5 %)
MET, EGFR, PDGFRA	Any CNV	2	3	0	26	31	6%	100.0% (15.8%,100.0 %)	89.7% (72.6%,97.8 %)	90.3% (74.2%,98.0 %)

Supplementary Table 15. Inclusion/exclusion criteria for clinical concordance study.

Inclusion criteria (all of the below)

- Patients must be 18 years or older.
- Patients with stage IV non squamous NSCLC who are either newly diagnosed or progressing on any treatment (progression defined as increasing tumor size or new metastatic lesions on clinical or imaging assessment). Patients with available tissue sample from a metastatic site or, if the patient presents with stage IV disease at diagnosis, from the primary tumor or a metastatic site. If a patient is progressing on EGFR targeted therapy (erlotinib, gefitinib, afatinib), tumor tissue sample is required only if available.
- No new systemic anti-tumor therapy administered in the interval between the tissue biopsy and collection of the blood sample (interval not to exceed eight weeks). Local radiation therapy is permitted.
- Able and willing to read, understand and sign an informed consent and Health Insurance Portability and Accountability Act (HIPAA) authorization where this is applicable, or equivalent in other geographies as required.

Exclusion criteria:

- Patients who are currently receiving therapy (targeted, immune therapy or chemotherapy) without sign of progression.
- Patients with squamous NSCLC.
- Patients with more than 8 weeks between collection of tumor specimen and collection of blood sample for analysis. (Not applicable for patients progressing on EGFR targeted therapy)
- Patients changing EGFR therapy due to toxicity or preference without documented disease progression.
- Patients progressing on osimertinib.
- Patients with brain metastases only.
- Patients who are unable to comply with study and/or follow-up procedures.
- Patients who are unable or unwilling to provide written informed consent.

Supplementary Methods

Supplementary Method 1: Analytical validation study design and statistical methods

Limit of blank (LOB): cfDNA samples from 20-40 mL blood from 103 individual healthy controls were collected in either Streck or Roche blood collection tubes and processed through the standard workflow using a range of DNA inputs (10-50 ng). The sample size exceeded the recommended minimum of 60 as described in CLSI EP17A, *Evaluation of Detection Capability for Clinical Laboratory Measurement Procedures; Approved Guideline*. In addition, 10 replicates of sheared/sized selected HapMap DNA were processed for input levels of 10 ng, 30 ng, and 50 ng. Within each variant type, LOBs were determined for individual variants or designated collections of variants (for example, SNV hotspots were divided into low background and high background). For any given collection, the maximum detection metric value across all member variants was computed for each sample and those values used to compute the LOB for the group.

Interfering substances: A variety of conditions were tested to ensure specificity in the presence of potential interfering substances. High-molecular-weight DNA (HMW-DNA) from buffy coat was spiked into cfDNA and sheared/size selected reference standard DNA at 4 levels (0%, 17%, 33%, and 100%). Stability of blood in both Roche and Streck tubes was determined using blood collected from healthy controls (8 tubes per donor) and held at room temperature for 0, 3, 5, or 10 days. The ability to detect cross-contamination was evaluated either prior to index ligation or after index ligation. In addition, the impact of cross-talk (or “bleed-over”) was evaluated by sequencing samples with an Indel at 25% allele fraction (AF) and a sample with an SV at 25% or 50% AF was sequenced with cfDNA samples from healthy controls.

Limit of detection (LOD): Study design was performed in accordance with CLSI *EP17A, Evaluation of Detection Capability for Clinical Laboratory Measurement Procedures; Approved Guideline*, which suggests inclusion of 60 low-level samples. Variant positive reference standards containing SNVs, indels, and SVs (Horizon DX) were sheared and size-selected in multiple batches and diluted into sheared/size-selected HapMap DNA to various AFs and run through the standard process as shown in Table 1. Digital droplet PCR (ddPCR) was used to verify achievement of targeted dilution levels prior to library preparation. For CNVs, LOD was established using pools of cell line DNA containing amplifications of *MET*, *EGFR*, and *ERBB2* as shown in Table 2.

A suitable *ALK* control was not available; thus it was not possible to establish LOD for the *ALK* fusion. However, during a physician experience program with version 1 of the assay, an *EML4-ALK* fusion was found at approximately 0.1% AF in an NSCLC patient with >900 ng cfDNA, and thus 10 replicates of this samples were run as part of the LOD study.

LODs were established using probit regression with input sample AF or copy number equivalent as the independent variable, and detection rate as the response. The study included dilutions of three preparations which were processed with three different lots of reagents, and LOD estimates were calculated separately for each reagent combination. Where possible, data for each individual variant were fitted separately. Otherwise, all the variants for a given variant type were pooled and a single curve fitted.

Accuracy: Blood samples from 36 cancer patients with known variant(s) in the tissue were purchased (Conversant Bio). ddPCR assays were designed to the variant(s) provided by vendor and either cfDNA or pond library (when insufficient residual cfDNA) tested. In addition, 25 different variant-positive cell line

DNAs (Horizon DX), a commercially purchased plasma matrix sample containing known variants, and a total of 15 HapMap variant negative controls (5 different HapMap samples, 3 independent sheared/size selected batches each) were tested. Since the variants reported by commercial vendors were listed by amino acid change, corresponding genomic coordinates, reference sequence, and variant sequences were identified for SNVs and Indels. Variants were considered detected if a variant found in the sample had a matching coordinate representation. Positive percent agreement (PPA) and technical positive predictive value (TPPV) were calculated as follows:

Positive percent agreement (PPA) = [A / (A+C)] * 100%

Technical positive predictive value (TPPV) = [A / (A+B)] * 100%

A = number where the assay is positive given that the variant is known to be present

B = number where the assay is positive given that the variant is known not to be present

C = number where the assay is negative given that the variant is known to be present

95% confidence intervals for the PPA and TPPV point estimates were calculated using the Jeffrey's and Clopper-Pearson methods.

Reproducibility and repeatability: Reproducibility and repeatability of the end-to-end assay (extraction to sequencing) was assessed using contrived samples. Variant positive reference standards containing a total of 11 SNVs, two Indels, three SVs, and one CNV gain were spiked into pools of plasma from healthy individuals. Variant positive reference standards were first diluted into HapMap DNA to obtain AFs representative of patient samples (0.72%-6%). The study spanned 3 weeks and included two adapter lots, two bait lots, three pond kit lots, and two sequencers. See Table 3 for the list of runs completed.

Supplementary Notes

Supplementary Note 1: Analytical validation results

Specificity: Specificity was established using data from the LOB and Interfering Substances Study. In the LOB, the signals from two variants (17:37872568:A and 10:43610128:T) in two different cfDNA samples appeared well above the background noise level and were anticipated to be true positives. ddPCR assays were therefore designed to these variants and verified. With the exception of these two variants and known SNPs, thresholds were set at or above the signal from all LOB samples, which represents >99% per-sample specificity.

Sensitivity: The limit of detection (LOD) of an assay is the lowest amount of target that can be detected by a test system with a stated probability (CLSI). The LOD95 (lowest amount of target for 95% detection rated) was determined for each variant type.

For SNVs, the reference standard material contained 10 SNVs verified to be present by the vendor (Horizon DX). Data for all SNVs within a batch of reference standard material were pooled and the LOD95 calculated. As shown in Supplementary Table 4, the LOD95 varied between batch (0.32-0.43 AF), which is not unexpected given these are point estimates derived from probit models. The pooled batch average was 0.37% AF.

For indels, the LOD95 was determined for the two most common Indels in NSCLC (*EGFR* 9-bp insertion and 15-bp deletion). The overall LOD95 for each Indel was similar (0.13% and 0.07% for insertions and deletions, respectively) with the overall batch average for Indels being 0.10% AF (Supplementary Table 5).

For structural variants, the LOD95 was determined for two translocations with clinical actionability in NSCLC: SLC34A2→ROS1 and CCDC6→RET. The reference standard control used to determine LOD was also shown to harbor the reciprocal ROS1 translocation (ROS1→SLC34A2), so the LOD95 was also calculated for this translocation. The overall LOD95 for each translocation was similar (0.37%-0.54%) with the overall batch average for SVs being 0.44% AF (Supplementary Table 6). Given that the EML4-ALK translocation is the most common translocation in NSCLC, it was also important to determine the sensitivity of the assay for this variant. In the absence of a suitable reference standard, a patient sample containing the EML4-ALK translocation was run a total of 10 times (starting from cfDNA). One library failed quality metrics prior to sequencing, but the EML4-ALK translocation was detected in all nine libraries sequenced at an AF of 0.15%-0.26%. A ddPCR assay was designed to this specific EML4-ALK translocation and verified to be present at 0.12%.

For CNVs, three samples were used to estimate the LOD95; one sample was a mixture of two cell lines, containing both an *EGFR* and *MET* copy number (CN) gain. The second sample contained a *ERRB2* CN gain, and the third contained a *MET* CN gain. Supplementary Table 7 represents the LOD95 point estimates for CNVs using these three samples.

Accuracy: Various types of samples were used to establish accuracy; blood from cancer patients previously shown to have a variant in their tissue, or detected by LSv1.0, variant positive and variant negative reference standards (sheared and size selected to represent cfDNA), and a plasma matrix sample containing known variants. The latter two sample types are representative "contrived" samples, which are

defined in the MolDX guidance (M00135) as "those created from controlled materials designed to define the analytical limits of the assay." Eighty-one samples passed the standard quality metrics and are summarized in Supplementary Table 8.

The following represented exclusions from analysis:

- No ddPCR results could be obtained for variant N1259K in cfDNA from cancer patient (ID#120338490), thus it was excluded.
- V769L in cfDNA from cancer patient (ID#120612937) was tested as negative by ddPCR; thus this variant was not included in the analysis because it was no longer an "expected variant" based on ddPCR data.
- Indel V237fs in sample HD786 is not targeted by the 17-gene panel and was therefore not included in the analysis.
- The commercially purchased plasma matrix sample failed a standard quality metric for low coverage and was excluded.
- No suitable ddPCR assay could be obtained for the *ALK* fusion in cfDNA from cancer patient (ID#120694318), therefore it was excluded.

PPA and TPPV are summarized in Supplementary Table 10.

There were two false negatives in this study (both SNVs), but these were both below the LOD95: G13D KRAS was found to be present in cfDNA at 0.16% AF by ddPCR but not by the 17-gene liquid biopsy assay. In addition, a de novo K117N NRAS which was present in stock cell line DNA at 50% AF was intended to be diluted to 1% AF. However, by ddPCR, this variant was shown to be at 0.07% AF and thus not detected by the 17-gene liquid biopsy assay.

Although there were two variants that counted as false positives in this study, one was likely a true positive. A purchased plasma sample was found to contain an *EGFR* Indel at 0.07% AF by the 17-gene liquid biopsy assay, and this matched the information provided by the vendor based on tissue testing. However, it was not found in the cfDNA by ddPCR, which was being used as the comparator/gold standard in this study. Therefore, the only probable true false positive in this accuracy study was a de novo Indel in *EGFR*, which was detected in one of three replicates in a NA24385 (HapMap) sample.

There were two other unexpected variants detected by the 17-gene liquid biopsy assay, but these were confirmed to be present by ddPCR, as shown in Supplementary Table 11.

Repeatability and reproducibility: All expected variants were observed, indicative of 100% PPA and reproducibility[1]. Supplementary Table 12 illustrates the frequency of observations as number of actual observations over number of expected observations.

---

[1] Positive Percent Agreement = PPA = Detections/(Detections+Failures to Detect)*100%
Reproducibility = (# of concordant expected variants)/[(# of concordant expected variants)+(# of discordant expected variants)]*100%

Interfering substances: The impact of high-molecular-weight DNA (HMW-DNA) was assessed with regard to variant specificity and sensitivity. As expected, 100% HMW-DNA failed to generate sufficient pond library to continue processing. Yields were below the limit of quantitation, demonstrating that HMW-DNA fragments are not incorporated into pond libraries. There was no increase in CNV signal, nor any false positive CNVs detected, with the addition of HMW-DNA, indicating that HMW-DNA does not appear to be an interfering substance in terms of specificity. All expected variants were detected with the exception of one CNV in one replicate, and one fusion in another with no overall trend with increasing amounts of HMW-DNA addition, indicating it is unlikely that HWM-DNA impacts the sensitivity of the assay at the levels tested.

Blood stability: There was no significant impact on pond yield or quality metrics after up to 10 days of blood storage for either tube type. There was no increase in CNV signal, nor any false detection of CNV. Blood storage for up to 10 days demonstrated no interference to assay performance.

Cross talk: All samples intentionally cross-contaminated (pre- and post-index ligation) were detected using the contamination module. No false positive calls as a result of crosstalk (i.e., bleed-over) were found when libraries containing 25% AF (Indel and SV) or 50% AF (SV) were sequenced with cfDNA from healthy controls.
